# How to Innovate as an Anesthesiologist: Guidance From the American Society of Anesthesiologists Committee on Innovation

**DOI:** 10.1213/ANE.0000000000008075

**Published:** 2026-05-18

**Authors:** Neil E. Rens, Nathan J. Smischney, Jiapeng Huang, Haobo Ma, Roya Saffary, Smitha Arekapudi, Ronald G. Pearl, for the American Society of Anesthesiologists Committee on Innovation, Andrew M. Bishara

**Affiliations:** From the 1 Department of Anesthesiology, Massachusetts General Brigham, Boston, Massachusetts; 2 Department of Anesthesiology and Critical Care Medicine, Mayo Clinic, Rochester, Minnesota; 3 Department of Anesthesiology and Perioperative Medicine, University of Louisville, Louisville, Kentucky; 4 Department of Anesthesia, Critical Care and Pain Medicine, Beth Israel Deaconess Medical Center, Boston, Massachusetts; 5 Department of Anesthesiology and Critical Care Medicine, Johns Hopkins University, Baltimore, Maryland; 6 Lakeview Anesthesia Associates, LLC, Chicago, Illinois; 7 Department of Anesthesiology, Perioperative and Pain Medicine, Stanford University School of Medicine, Stanford, California.

## Abstract

While the field of anesthesiology has a storied history of innovation, especially related to patient safety, contemporary anesthesiologists face growing barriers to innovation from complex regulatory requirements and financial constraints. The Biodesign framework offers a systematic approach to medical technology development that can be applied to the perioperative space to address these challenges. This process begins with identification and screening of high-value clinical needs, followed by generation and evaluation of potential solutions, and culminates in strategy development, business planning, and fundraising. Each stage is designed to guide clinicians from early observation through translation into viable technologies. By adopting this structured method, anesthesiologists are well-positioned to transform clinical insights into scalable solutions, ensuring the specialty continues to advance patient safety and care in the face of evolving clinical demands.

Innovation is foundational to the field of anesthesiology and its practice. Although anesthesiologists have been innovating since the dawn of the field, increasing regulatory and financial complexity has dramatically changed how clinicians create new innovations. The American Society of Anesthesiologists Committee on Innovation has received increasing requests for guidance about how anesthesiologists can be successful innovators. Extensive literature already exists on innovation broadly and healthcare innovation specifically.^1–3^ Additionally, others have published about past innovations in anesthesiology and the need for future ones.^4^ However, there is a paucity of literature on how to innovate within the field of anesthesiology. Here, we summarize the Biodesign process,^5^ one of the most widely cited frameworks for biomedical innovation, and apply it to the field of anesthesiology. Biodesign is a structured approach that starts with identifying a need, creating concept solutions, and culminates in business planning (Figure 1).

## NEEDS FINDING

Using a robust process for “Needs Finding” ensures that the final solution addresses an important problem. As perioperative physicians, anesthesiologists frequently encounter problems and have an intuitive sense of possible solutions. Needs Finding helps innovators—especially those entrenched in the problem as key stakeholders—to pause and consider all facets of a problem.

**Figure 1.**
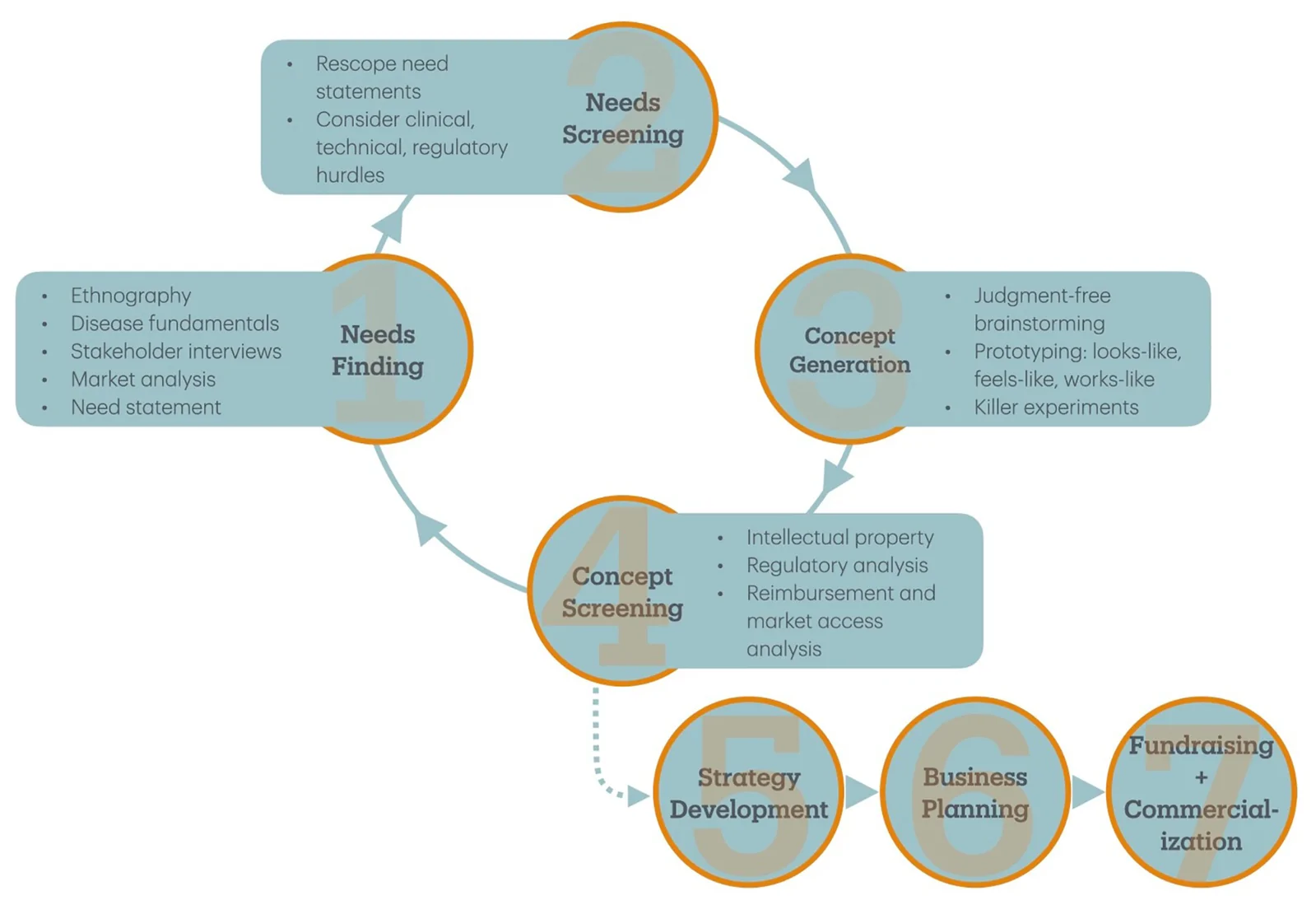
The Biodesign process involves identifying unmet needs, inventing solutions, and implementing them. Innovators benefit from a funnel approach where many needs are identified and filtered to the most promising ones. Then many possible solutions are brainstormed but are also filtered to the single one most likely to succeed. The process is highly iterative and innovators may return to the Needs Finding phase after discovering a solution to the previously identified need is not feasible. Once ready for implementation, innovators develop a business strategy and business plan in preparation for fundraising. Adapted from Biodesign: The Process of Innovating Medical Technologies.^5^

Needs finding involves four steps.

1. Identify pain points through ethnography—immersion into an environment to observe and learn.
2. Understand the relevant epidemiology, anatomy, and physiology.
3. Analyze the entire cycle of care, including upstream factors leading to the problem and what downstream consequences result from the problem.
4. Perform a rudimentary market analysis by estimating the number of patients affected and related costs.

The iterative needs finding process allows innovators to generate a “Needs Statement.” which frames pain points as, “A way to [address the problem] in [a specific population] in order to [achieve a desired outcome]” (Figure 2). Needs Statements should specify the efficacy, cost, safety, and usability required for an innovation’s success. These need to be broad enough to encompass a significant and compelling market but focused enough to be actionable. They should center on a single outcome that can be objectively measured. Most importantly, they must be “solution-agnostic.” Specifying a particular solution in the statement artificially constrains innovators when they arrive at the prototyping stage.

**Figure 2.**
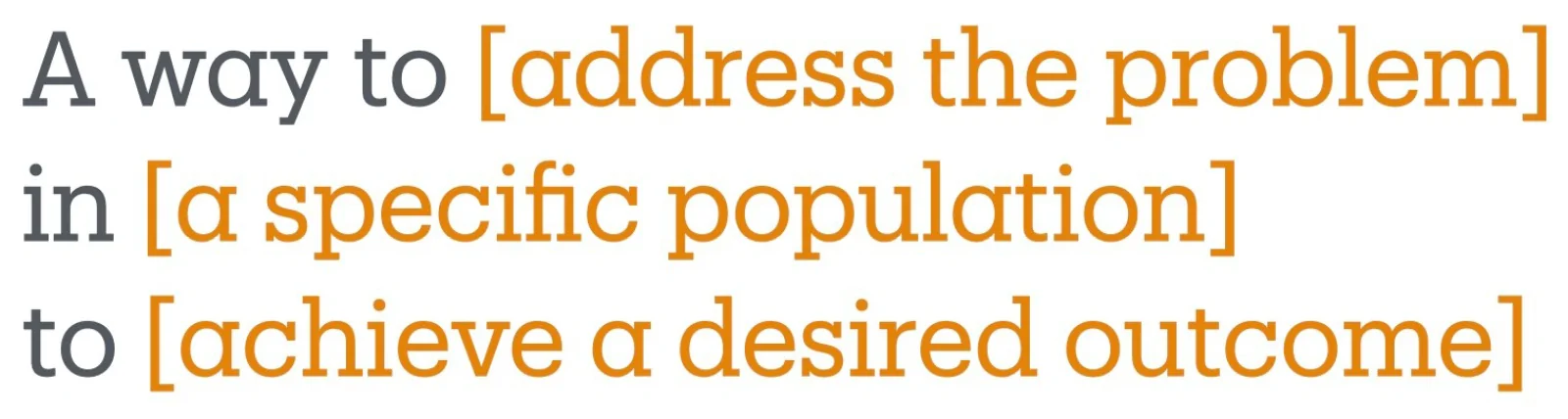
The Need Statement is a tool to help innovators identify and frame unmet needs. The scope can be adjusted to be a broad population, for example, “patients with hypertension,” or a narrow one, for example, “female patients with secondary hypertension complicated by left ventricular hypertrophy.” Importantly, Need Statements should be “solution agnostic” so they do not artificially constrain brainstorming by implying a particular solution. However, specifying a desired outcome within the Need Statement is critical to guiding the brainstorming process. Adapted from *Biodesign*.^5^

Consider the cardiac surgery intensive care unit (ICU), home to some of the highest-risk patients. In the 1980s, infusions in the ICU were delivered via bulky, non-portable mechanical syringe pumps. Moving to the operating room (OR) required the anesthesiologist to mix new dilutions bedside in the ICU. Errors frequently occurred, and multiple patients suffered. Collaborating with engineer Dean Kamen, Massachusetts General Hospital anesthesiologist Nat Sims developed a portable, programmable infusion pump that did not require mixing different dilutions to deliver different doses.

Although this innovation predates literature about the Needs Statement, innovators at that time were likely considering something like: Anesthesiologists and ICU nurses need a way to [quickly and safely titrate dangerous, life-sustaining medications] in [unstable cardiac surgery patients] in order to [reduce the time it takes to travel from the ICU to the operating room]. They spent significant time upstream of the “anesthesia” problem to understand the ICU infusion needs. This stakeholder engagement was critical in driving adoption across the entire cycle of care. Finally, note that the statement is solution-agnostic; nowhere does it specify that the solution should involve syringe pumps.

In summary, Needs Finding provides a structured process for identifying and refining problems. Anesthesiologists encounter problems daily, but sometimes it is unclear which problems merit larger-scale solutions. When thinking about high-value need areas, successful innovators focus on shortening length of stay, keeping patients away from higher levels of care, reducing procedure time, decreasing resource use, and decreasing complications. With numerous Needs Statements in hand, including multiple statements scoping the same problem in different ways, they are ready to move to “Needs Screening.”

## NEEDS SCREENING

In the Biodesign process, Needs Screening serves as a critical gatekeeping step that ensures that only the most impactful, tractable, and unmet clinical needs are pursued for solution development. This phase occurs after initial clinical immersion and Needs Finding where a broad set of potential problems are observed and documented. The purpose of screening is to prioritize these needs using a structured, analytical framework that evaluates each need’s clinical impact, market potential, existing solutions, technical feasibility, and alignment with stakeholder interests. Needs Screening transforms a long list of problems into a short list of opportunities by applying criteria such as the prevalence and severity of the condition, current standards of care, technological barriers, reimbursement pathways, and regulatory complexity. Crucially, this step prevents wasted effort on needs that may seem promising but ultimately lack clinical urgency, scalability, or development viability.

As discussed previously, the realm between the cardiac surgery ICU and OR highlights the importance of rigorous needs screening. Clinically, the problem was common in high-acuity cardiac procedures and directly impacted patient outcomes, making the need highly significant. Existing solutions were fragmented—relying on disconnected pumps and manual mixing—and did not offer synchronized control or prioritization of infusion protocols. The team found strong stakeholder alignment: anesthesiologists, intensivists, perfusionists, and ICU nurses all experienced similar challenges. Market analysis revealed that while infusion pumps were a mature market, there was a growing need for specialized intelligent delivery systems without need for interruptions and tailored to high-risk environments such as ORs and ICUs. Technically, it was feasible to design a programmable pump system with integrated algorithms that could co-infuse multiple drugs, adjust doses based on user-defined protocols, and provide visual alerts without interruption between environments.

As a result of this comprehensive Needs Screening process, the team greenlit solution development. The cardiac anesthesiologist led the design of a programmable “smart” infusion pump that in future iterations included a drug library with recommended dosing and infusion rates. The prototype integrated user interface, safety interlocks, and software algorithms. The project attracted institutional interest and advanced through early bench testing and usability studies. This case underscores that Needs Screening in Biodesign is not just about filtering ideas—it’s about selecting the right clinical challenge that, when addressed, can lead to scalable life-saving technologies.

## CONCEPT GENERATION

Concept Generation is the structured process of validating an idea in a goal-directed manner, with emphasis on both value and novelty. The first step involves brainstorming to produce as many ideas as possible without concern for quality or plausibility. In this creative stage, there are no “wrong” ideas. Teams are ideal for fostering creativity, and productivity is maximized when participants defer judgment, encourage bold thinking, build upon each other’s ideas, prioritize quantity over quality, engage in one conversation at a time, and use visual tools.^6^ Collaborating with university engineering programs can be a powerful partnership; anesthesiologists can bring clinical knowledge and clinical needs while engineering professors and students provide technical prototyping skills. Artificial Intelligence (AI) has the potential to be a powerful tool in Concept Generation. It can extract insights from both unstructured data and structured databases and categorize what innovations have already been tried and why they failed. AI’s “Achilles’ heel” - hallucination - can actually be useful in creative brainstorming.

*Prototyping*: After identifying the most promising solutions, prototyping is the next step in the Biodesign process. The goal is to translate a promising concept from an idea into a preliminary design and ultimately into a working form. Early-stage prototyping must focus on simple, inexpensive, and quick processes to allow for fast iterations and identify possible bottlenecks in the product development process. The process of prototyping is a highly iterative endeavor, where each successive prototype should answer critical questions about the device and its functions. Different questions demand different types of prototypes, ranging from “looks-like” prototypes to “feels-like” to “works-like.” This allows for improvement of the design while addressing deficiencies in the product. As the project progresses, the technical requirements, designs, and models call for more robust prototypes. Refinement of functionality and features requires a combination of user, bench, simulated use, and tissue testing to comprehensively meet essential design requirements.^6^

*Functional blocks*: The ability to break down the selected concept into smaller functional blocks is critical to developing the prototype in a focused and iterative manner. A functional block can be defined as a subset of principles, skills, technologies, and expertise needed for the solution to perform a characteristic function. Many functional blocks can be identified for systems and equipment in anesthesiology; for example, the anesthesia workstation can be divided into three functional blocks: gas delivery, mechanical ventilation and monitoring systems. Selecting functional blocks appropriately allows for evaluating different processes and elements of the envisioned solution without needing a working prototype beforehand. Moreover, addressing functional blocks individually optimizes the time, resources, and skills required.

*Derisking* refers to the active process of identifying and addressing possible risks that can be encountered while developing the prototype. Innovators should focus on prototyping a specific functional block with straightforward questions in mind. Derisking and functional blocks are two intrinsically related concepts: functional block identification helps answer specific questions to remove risks of failure, and derisking helps identify functional blocks. Through this iterative process, innovators can quickly identify which functions of a concept can or cannot be realized, speeding up the product development stage by moving forward with promising solutions and discarding others.

For example, early studies documented the limitations of traditional clinical signs as surrogates for anesthetic depth.^7^ Advances in neuroscience and EEG spectral analysis revealed correlations between brain electrical activity and levels of consciousness.^8,9^ Biodesign teams conceptualized a device that could transform raw EEG signals into a dimensionless index of hypnotic state (BIS monitor), filling a critical unmet need for real-time, objective monitoring of anesthetic depth. Natural functional blocks for BIS included sensor design and algorithm development. Derisking likely focused on early experiments to see if an algorithm could distinguish between conscious and unconscious states.

## CONCEPT SCREENING

Concept Screening is the structured evaluation of early-stage innovations to identify those with the greatest potential for clinical value and feasibility, narrowing a broad set of ideas into a smaller group of promising candidates for further development. Concept Screening is used to compare alternative solutions and select those with the highest real-world potential, thereby conserving resources and focusing attention on concepts with a realistic path to clinical benefit, regulatory approval, and adoption.^5^ To justify advancement, a concept must show potential to improve patient care, such as enhancing safety, decreasing complication rates, or supporting faster recovery. In addition, any proposed innovation must be technically feasible, meaning the underlying science, engineering, or digital infrastructure can realistically support prototyping, validation, and eventual clinical implementation.

Once clinical value and technical feasibility are established, regulatory and market conditions must be assessed. Intellectual property (IP) determines whether the concept is patentable—novel, non-obvious, and useful—and whether freedom to operate exists in a crowded patent landscape.^10,11^ Regulatory assessment establishes how the innovation will be reviewed by the FDA, including its likely classification as Class I (low risk), II (moderate risk), or III (high risk)—and whether it will follow a 510(k) pathway (substantial equivalence to an existing device), seek De Novo authorization, or require a Premarket Approval.^10–13^ Reimbursement and market access are equally critical. A technology that is clinically effective may still fail without a mechanism to cover its costs. Key questions include who will pay—insurers, hospitals, health systems, or the industry itself? While formal cost-effectiveness analyses are typically performed later, Concept Screening should establish whether a technology has a plausible path to cost-effectiveness, such as preventing complications or reducing downstream resource use.

The BIS monitor illustrates how a first-in-class innovation advanced successfully through this stage. BIS provided a novel solution for monitoring anesthetic depth by converting EEG signals into a processed index. Technically, BIS was supported by advances in EEG signal processing that enabled real-time calculation of bispectral parameters. From an IP standpoint, BIS was protected through patents. Regulatory feasibility was favorable: BIS was classified as a Class II device and achieved FDA 510(k) clearance. On the reimbursement side, BIS adoption was complicated by the absence of a dedicated billing code. The manufacturer addressed this barrier through a “razor-and-blade” business model in which revenue came from disposable sensors, along with licensing agreements that integrated BIS into monitors from manufacturers.

Initial enthusiasm for using BIS monitoring to reduce intraoperative awareness was dampened following the BAG-RECALL trial.^14^ Subsequent efforts to apply BIS monitoring to reduce postoperative delirium were also not consistently successful.^15,16^ However, BIS monitoring may facilitate faster emergence and extubation.^17^ Collectively, these findings underscore that innovation requires clinical testing and alignment with measurable outcomes.

## STRATEGY DEVELOPMENT

The Strategy Development phase shifts the focus from “Can we build it?” to “How should we position it?” to move an innovation into clinical use and eventually to market. A strong strategy concentrates resources on the most promising initial application and creates a clear path toward long-term impact.

A key concept is identifying a “beachhead market.” The term, which originated from the World War II Battle of Normandy, refers to a narrow target market segment most likely to succeed.^18^ Selecting this target requires balancing technical feasibility, unmet clinical need, regulatory challenges, reimbursement, and the likelihood of enthusiastic early adopters. For anesthesiologists, the beachhead approach focuses on the segment with the clearest pain point and the fewest barriers to adoption. For example, a device designed to monitor perfusion could, in theory, be useful across multiple surgical specialties. However, the innovation team can identify specific surgeries, such as liver transplantation or cardiac surgery, as the ideal initial segment where providers are acutely aware of perfusion challenges and willing to try new tools. By demonstrating value in these initial use cases, the innovation gains credibility, allowing for expansion into broader perioperative settings.

Key questions to ask when defining a beachhead market include:

1. Which patient populations are most affected?
2. Who is most motivated to adopt new solutions?
3. How does the innovation align with current reimbursement pathways?
4. Can clinical and economic value be clearly demonstrated?

Along with the beachhead strategy, innovators should focus on protecting their IP. An effective IP strategy protects the innovation from competitors while allowing flexibility for partnerships and commercialization. It can also be used as a strategic tool to attract investors and ensure growth.^19^ When developing an IP strategy, it is important to consider both patentability and freedom to operate. For a patent to be granted, an innovation must be novel, non-obvious, and useful.^20^ However, even if a patent is possible, innovators should complete a freedom to operate analysis to ensure that their innovation is not infringing on existing patents. For anesthesiologists, IP strategy may involve devices, methods of use, software algorithms, drug formulations, or synthesis and manufacturing methods. Strong IP is not limited to a single patent and should include creating protections around key aspects of the solution (eg, hardware design, user interface, data processing methods). Once filed, IP can still be an expensive budget item due to patent defense and litigation. Engaging institutional technology transfer offices early can help navigate these complexities and potentially help with funding.

Beyond the beachhead market and IP, innovators must also consider regulatory pathways, reimbursement strategies and commercial models. The nuances of regulation and reimbursement are outside the scope of this article but are described at length elsewhere.^13^

Case Example: Strategy for portable perfusion monitoring device

- Beachhead Market: Ambulatory surgical centers and high-risk ORs, where early detection of hypotension and poor perfusion can prevent complications and unplanned admissions.
- IP: Patents may cover novel wireless sensor capabilities and cloud-based analytics for continuous perfusion and blood pressure monitoring. Freedom-to-operate analysis confirms the combination of portability and real-time data is unique.
- Regulatory Pathway: Other noninvasive devices exist that monitor blood pressure and perfusion; therefore, a 510(k) submission can be filed which will reduce time and cost.^21^
- Reimbursement Strategy: Current intraoperative monitoring billing codes apply. Cost savings occur through reduced complications, shorter length of stay, and fewer ICU transfers.
- Commercial Model: Direct sales to surgical centers with a subscription-based cloud analytics platform creating recurring revenue. Later expansion targets hospital systems and international markets.

Strategy Development bridges the gap between promising prototypes and successful adoption. By focusing on a well-defined beachhead market, protecting IP, and aligning regulatory, reimbursement, and commercial strategies, anesthesiologists can increase the likelihood that their ideas move beyond the laboratory into widespread clinical use.

## BUSINESS PLANNING

Business Planning prepares anesthesiologists to transition to a sustainable business model through operational planning, budgeting, financial projections, and market analysis. One of the first critical decisions is determining the right business strategy: should they license the device to an established company, start a new venture, or form a partnership with a medical device firm? Additionally, defining the revenue model is essential—will it be based on direct sales, subscriptions, disposable components or something else? A detailed business plan should cover a wide range of elements, from cost structures and manufacturing needs to pricing strategies, reimbursement models, and revenue projections.

Business Planning is crucial throughout the development and commercialization process. Development of a medical device typically spans years and requires coordination across multiple teams, including medical, engineering, regulatory, and business domains. Tools like a Gantt chart are invaluable for managing timelines and tracking milestones. A Gantt chart provides a visual representation of the project, showing when different tasks need to be completed. It helps teams identify dependencies between tasks, allocate resources efficiently, and communicate progress to investors and stakeholders.

A well-crafted budget is essential for ensuring efficient resource allocation throughout the development process. A typical medical device launch may require millions of dollars over 30 months. The budget must cover all expenses, including product design, regulatory affairs, preclinical testing, patent attorneys, and marketing personnel. Financial projections should include revenue models to support continuing development of the device.

## FUNDRAISING AND COMMERCIALIZATION

Fundraising is the final step in moving Biodesign innovations into successful commercialization. It builds upon the earlier stages of the Biodesign process. The fundraising process begins with creating a pitch deck, including a financial model that provides an operating plan, staffing structure, cost projections, cash flow statement, market model, and income statement. The pitch deck is tailored to the specific target audience, addressing risks and opportunities.

Fundraising can occur at multiple stages, from concept development and prototyping to regulatory approval, commercialization, and growth. Early-stage investments typically carry the highest risk for funders, leading to higher costs per dollar of funding. However, early stages require less capital than later stages when costs escalate due to clinical trials, regulatory requirements, and reimbursement planning. Funding can be considered in four stages:

1. Seed funding: Supports concept development, prototyping, and feasibility studies.
2. Start-up funding: Facilitates early clinical trials and initial commercialization efforts.
3. Expansion funding: Helps fund clinical trials, regulatory approval, and early-stage commercialization.
4. Mezzanine funding: Supports rapid growth and market expansion.

Total cost to progress from concept to profitability or acquisition often exceeds $100 million.

Funding can be dilutive, where investors own equity in the company, or non-dilutive, which comes in the form of debt or grant funding. Venture capital (VC) firms are a common source of dilutive funding and provide substantial capital, but they typically expect high rates of return so may not be interested in early-stage ventures. VCs may offer valuable experience and networking opportunities, but may also demand significant control. VC firms generally expect to exit within five years through an Initial Public Offering (IPO) or acquisition. Angel investors are similar to VCs but often frequently support earlier-stage ventures and may be driven by the social impact of the innovation. Corporate investors may provide funding when the innovation aligns with their products or business goals.

Non-dilutive funding may or may not require repayment. In debt funding, the entrepreneur borrows capital with the requirement to repay. Grant funding, typically provided by government agencies, foundations, or corporations, does not require repayment. The Small Business Innovation Research (SBIR) and Small Business Technology Transfer (STTR) programs support early-stage technology development, with Phase I awards up to $314,363 and Phase II awards up to $2,095,748.^22^ Foundations and societies offer funding that does not require repayment. Academic institutions provide competitive early-stage grants, but the resulting IP is shared between the individual and the institution. University technology licensing offices assist with patent protection, commercialization, and license agreements.

In the early phases of development, entrepreneurs may rely on self-financing, loans from friends and family, or crowdfunding to secure initial capital. Incubators provide infrastructure, mentorship and networking opportunities, but may charge for their services. Philanthropy can provide funding for early-stage development, particularly for projects with a social or clinical impact. Finally, at any stage in development, entrepreneurs may consider licensing or selling their technology as an alternative to raising capital.

As an example of Business Planning and Fundraising, Leaf Healthcare was a company founded by two Stanford medical students to address the problem of hospital-acquired pressure injuries (HAPIs). They recognized that HAPIs occur due to high pressure over an extended period of time. The team developed a lightweight, wireless sensor worn by patients to monitor position and movement, providing real-time feedback to support adherence to evidence-based turning protocols. They developed a needs statement, defined the target population, the key stakeholders, and desired outcomes (improved quality of care producing decreased HAPIs, decreased readmissions, decreased length of stay, and increased workflow efficiency). Early-stage costs were minimal and were self-financed. They created a business plan that included vision, strategy, and financial projections. Between 2011 and 2015, they obtained several rounds of funding from angel investors to support prototyping, regulatory filings, incorporation, and early clinical trials. They received FDA clearance in 2013, followed by commercial launch in 2014. They completed a pivotal randomized controlled trial in 2016 that demonstrated a 73% reduction in pressure injuries.^23^ Following publication, Leaf was acquired by Smith+Nephew. Subsequent studies confirm that the technology decreases HAPIs and is cost-effective. Leaf has been adopted by hospitals across the United States and is used on hundreds of thousands of patients.

## CONCLUSIONS

Following a structured innovation process like Biodesign can help anesthesiologists translate their clinical insights into meaningful innovations that improve patient care. The innovation framework presented here may appear linear but primarily is iterative. Anesthesiologists may start their journey through “user innovation” by organically prototyping solutions for their daily practice. Innovators may progress from Needs Finding to Needs Screening and into Concept Generation before realizing they need to adjust or even completely overhaul their Needs Statement. Incorporating lessons from Biodesign can help avoid common pitfalls when thinking about scaling. Biodesign helps ensure that the problem identified exists outside of one’s immediate practice environment, that the solution addresses the core underlying problem rather than a symptom of it, and that the innovation has a pathway to regulatory approval, IP protection, and reimbursement.

As anesthesiologists navigate the Biodesign process, AI presents new opportunities and challenges. It can be a powerful component of a solution, for example helping identify difficult airways or predict hypotension.^24,25^ However, it also has a role in the Biodesign process itself such as summarizing large volumes of qualitative data to help uncover challenges. The utility of AI is highly dependent on the quality of input data provided by innovators, and AI models still pose risks like bias, necessitating careful human oversight.

In conclusion, anesthesiologists are uniquely positioned to drive innovation. Their deep clinical expertise and broad exposure to different surgical and procedural interventions allows them to identify areas where new technologies can make meaningful improvements to patient care. However, success in this field requires more than just clinical insight; it also requires a structured, strategic approach to identifying problems, honing solutions, and navigating the world of business planning, financial management, and regulatory compliance. Biodesign offers a framework to help anesthesiologists move from pain point to product, improving patient care and transforming clinical practice.

## DISCLOSURES

**Conflicts of Interest:** J. Huang is a paid consultant to Medtronic, Accuryn Medical, GE Healthcare Technologies, Inc., and Grifols. No other authors declared Conflicts of Interest. **Funding:** None. **This manuscript was handled by:** Charles Emala, MS, MD.

## ACKNOWLEDGMENTS

This is a work product of the American Society of Anesthesiologists (ASA) Committee on Innovation. ASA staff were not involved in the writing of this article, and this paper is not endorsed by the Board of Directors or the House of Delegates.
